# Predictors of doping intentions, susceptibility, and behaviour of elite athletes: a meta-analytic review

**DOI:** 10.1186/s40064-016-3000-0

**Published:** 2016-08-11

**Authors:** Cornelia Blank, Martin Kopp, Martin Niedermeier, Martin Schnitzer, Wolfgang Schobersberger

**Affiliations:** 1 Institute for Sports Medicine, Alpine Medicine & Health Tourism, UMIT, Eduard-Wallnöfer-Zentrum 1, 6060 Hall in Tyrol, Austria; 2 Department of Sport Science, University Innsbruck, Fürstenweg 185, 6020 Innsbruck, Austria; 3 Tirol Kliniken GmbH, Innsbruck, Austria

**Keywords:** Doping prevention, Micro-level, Macro-level, Sporting culture

## Abstract

Research in doping has focused on potential intervention strategies, increasingly targeting predicting factors. Yet, findings are inconsistent, mostly athlete-centred and explain only limited variances in behaviour. This critical review aims to (a) summarize studies that identified predictors of doping intentions, susceptibility, and behaviour in elite athletes and to (b) analyse in how far previous research included aspects beyond athlete-centred approaches, such as context and sporting culture. We reviewed 14 studies that focused on elite athletes. Situational temptation, attitudes, and subjective norms seem to be strong predicting variables of doping intentions (*r* ≥ 0.50), but intention was no predictor for behaviour. Attitudes were a significant predictor for both, doping susceptibility (*r* = 0.47) and behaviour (*r* = 0.30). Most of the predictors are athlete-centred and ignore macro-level factors that might help to explain how certain individual traits impact on the decision making process. The findings from this review call for a critical discussion of whether current doping-prevention research needs to take new directions. We propose future research to bridge findings of psychologists and sociologists, as it appears that doping behaviour cannot be explained by ignoring the one or the other. Impacts of sporting culture that have been identified in qualitative approaches need to be integrated in future quantitative approaches to test for its external validity. Inclusion of both, micro- and macro level factors may enable an integrative prevention program that creates a sporting culture without doping.

## Background

Doping is generally considered to be unsportsmanlike and believed to create unfair advantages while destroying the values of sport. In view of the importance to protect the athletes’ health and integrity, results of a previous survey indicated that preventing doping in elite sport is considered as the highest priority from 35 international sporting federations (Mountjoy and Junge 2013). Nevertheless, Wagner and Pedersen (2014) have previously reported that the general population does not trust the doping management of international sporting federations due to ongoing doping scandals despite the implementation of the World Anti-Doping Agency (WADA) in 1999. Significant amounts of money have been spent on (a) identifying new policies and measures to prevent doping and (b) implementing these measures. However, if the general population perceives this money to be spent on inefficient prevention strategies, then their trust may decrease even further. Therefore, the identification of processes that lead to doping behaviour should be of interest to both science researchers and sport governing bodies entrusted with doping prevention. Prevention strategies that are grounded on transparently accumulated scientific evidence might help to reduce the above mentioned lack of trust not only of the general population but also of the athletes themselves (Overbye 2016).

## Deterrence and education

Increasing doping controls as part of a deterrence approach represents one possibility to prevent doping. Nevertheless, results from current research offer a number of explanations as to why this approach does not seem to be successful on its own. For example, as already mentioned above, there is a lack of trust among athletes in effective doping controls (Overbye 2016). Trust of the athletes is crucial for bans to work as a deterrent, as it is not the act of doping but the discovery of doping that is punished (Petróczi and Haugen 2012). Highlighting the negative health effects of doping has also been unsuccessful (Engelberg et al. 2015; Huybers and Mazanov 2012; Probert and Leberman 2009; Schnell et al. 2014). The only deterrents that appear to be partially effective are social sanctions and humiliation (Huybers and Mazanov 2012; Overbye et al. 2014), which were both included in the Australian Anti-Doping Agency’s prevention program called “You can never win your reputation back” (Huybers and Mazanov 2012). Based on this evidence, current doping prevention measures focus on more than mere deterrence strategies. Additional education-based prevention approaches (i.e., Goldberg’s ATLAS program) (Goldberg et al. 2000) have been increasingly applied to prevent negative behaviour before it occurs, especially with respect to athletes’ health, integrity, and fairness, as well as values in sports. Nonetheless, education in the sense of transferring information does not appear to be successful either. Previous studies have identified only weak, if any, association between knowledge about doping and its side effects, and doping intentions and/or behaviour (Blank et al. 2014; Ntoumanis et al. 2014). It appears that the effectiveness of the health message in trying to prevent doping is questionable (Engelberg et al. 2015). As a result of the acknowledged complexity of the doping phenomenon scientists suggest that only a firm understanding of factors involved in doping as well as their relationships will potentially result valid pro-social interventions for doping (Johnson 2012). Therefore, subsequent research that has focused on identifying reasons for doping behaviour, has been inspired mainly, but not exclusively, by research in the field of health- and social psychology (Lazuras et al. 2015) and focusing on the individual athlete.

## Psychological factors

Doping is considered to be a complex behaviour. A recent meta-analysis by Ntoumanis et al. (2014) summarized and compared literally all known psychological predictors of doping behaviour in all physical settings and at all performance levels. Findings included variables from the theory of planned behaviour (TPB) (Ajzen and Madden 1986), additional attitude-behaviour relations (Bentler and Speckart 1979), deterrence (Paternoster 1987) and self-determination theory (Ryan and Deci 2000), as well as combinations of these variables (Donovan et al. 2002; Strelan and Boeckmann 2003). In addition, implicit measures were also included (Brand et al. 2014; Petroczi et al. 2008). Even though this meta-analysis provides extensive information for the scientific and practical community, including all competition levels might dampen the significance of the result for the elite athletes. A number of studies included within this meta-analysis involve recreational athletes who are not part of sporting organizations that signed the WADA anti-doping code and are therefore not directly confronted with the offense of doping (Arandjelovic 2015). Additionally, motivations for doping in these sports may be different from motivations of elite athletes who are training to compete in major sporting events, such as the Olympic Games or World Championships (Bilard et al. 2011; Elliot and Goldberg 1996; Wiefferink et al. 2008). The Olympic Games are considered to be the most important event in an athlete’s life, and winning a medal at the Olympics is the highest goal to which an athlete can strive. Chester and Wojek (2015) have recently criticized existing research in recreational athletes as not necessarily being representative for elite athletes. It is expected that elite athletes face different situational pressures within their daily training routine, and a previous study has shown that situational factors mediate several predictors of doping behaviour (Barkoukis et al. 2013). In line, only a few of the findings from Ntoumanis et al. (2014) were observed in a recent qualitative study by Engelberg et al. (2015), who analysed interviews with doped athletes. Some correlations observed in the meta-analysis of Ntoumanis et al. (2014) were in the opposite direction of correlations observed in Engelberg et al. (2015). Explanations for these diverse findings might be (a) the different target populations, (b) difficulties of evaluating doping behaviour (i.e. in most research athletes are asked to self-report about their doping behaviour) and (c) different methodological approaches that are hardly comparable.

## Sociological approaches

Given the constant number of positive doping samples, one could either speculate that despite the growing body of research that helps understanding the underlying psychological processes of doping behaviour, the preventive strategies seem to lack success or that the analytical detection methods have improved. Considering the first hypothesis, many researchers opened the debate to shift from an athlete-centred approach to a much wider approach. Especially in sociology this debate already has an extensive history. Stewart and Smith (2008) have proposed a macro model that also includes sporting context and—culture. They further acknowledged that an athlete’s decision to dope might not always be rational and be influenced by a range of impacts. Therefore, even though beneficial, socio-psychological theories to explain doping behaviour might not always fit as they are mostly based on rational and intentional decision making. It has to be acknowledged that athletes are embedded in community cultures and practices (Wagner 2010). Copeland and Potwarka (2016) claimed that doping can be understood on a cognitive level of the individual, but furthermore that the contextual-organizational level must not be ignored. Parts of this contextual-organizational level are for example impacts of commercialization and globalization as well as sporting culture and the perception of its own identity (Stewart and Smith 2008).

Summarizing, there already exists a rapidly growing body of research aimed at explaining doping behaviour. Nevertheless, findings are very heterogeneous and appear to have a mainly athlete-centred focus which might be due to several reasons outlined above. This heterogeneous landscape of research findings renders the formulation of clear prevention strategy difficult and there is the need to summarize these findings to possibly identify generalizable common predictors.

## Aims and objectives

Building on the comprehensive work of Ntoumanis et al. (2014), this meta-analytic review has two major objectives. First, this review aggregates and interprets research efforts towards the identification of predictors of (a) doping behaviour, (b) intentions that are most proximal to doping behaviour (Armitage and Conner 2001; Bilic 2005; Elliot et al. 2003; Godin and Kok 1996), and (c) susceptibility to doping behaviour, which is commonly used as a substitute for doping behaviour itself. Given the expected differences between amateur and elite athletes that have been outlined above, only studies that included applied multivariate analyses of elite athletes competing at the national level or higher are included in this review. Findings based on different empirical models are reviewed and compared as appropriate. With this approach, we aim to determine whether it is possible to identify common predictors of doping intentions, susceptibility, and behaviour of elite athletes. More importantly, we aim to critically discuss whether it is reasonable to quantify overall effect sizes of these predictors due to different operationalization methods of equal psychological constructs.

The second major aim is to analyse whether or not previous research, that allows meta-analytic calculations, included aspects beyond the athlete-centred approach. Findings and possible non-findings will be critically discussed to provide both, a scientific data compilation that might allow for the definition of preventive strategies as well as starting points for future research to close a potential gap between micro- and macro level oriented research approaches.

## Methods

### Search methods for identification of articles

A systematic literature research was performed to document the findings of previous studies with respect to predicting factors on doping, intentions, susceptibility, and behaviour. Our search included original studies published in scientific peer-reviewed journals between 1999 (the founding year of WADA) and January 2016 and indexed in the MEDLINE and/or EBSCO (including SocIndex, Academic search elite, Business source premier, Cinahl, Pre-Cinahl, Hospitality and Tourism index, Inspec, PsychArticles, PsychInfo, SportsDiscus, Lista) databases using the search terms “doping”, “performance enhancing drugs”, “drugs AND sport” as well as by combining each of these terms with “determinants”, “correlates”, “risk factors”, “precipitating factors”, and “model”. This search term strategy was previously used by Backhouse et al. (2007) for a final report to the WADA. Additionally, we manually searched the reference lists of every primary study for additional publications.

### Assessed outcomes

The main outcomes of interest were predicting factors for doping susceptibility and doping behaviour; however, as intentions have been said to be an important proximal factor to behaviour (Armitage and Conner 2001; Bilic 2005; Elliot et al. 2003; Godin and Kok 1996), we included predicting factors for intentions as the third outcome of interest. Outcomes were exclusively recorded by self-reporting questionnaires and were displayed in prevalence percentages and/or computed scores as the results of regression analyses and/or structural equation modelling.

### Data extraction

Two researchers (CB, WS) independently performed the literature research, quality assessment, and data extraction. Any disagreements about inclusion of trials were resolved by discussion with the three remaining researchers (MK, MN, MS). According to a standardized form, the investigators extracted data that was methodologically and scientifically sound and collected the following variables: author and year of publication, journal title, characteristics of the target population of the study (including sample size and age), the dependent variable(s) of the study (intentions, susceptibility, behaviour), the included psychological concepts, and the outcome of the study (tested model and/or individual predicting factors).

### Inclusion and exclusion criteria

**Fig. 1:**
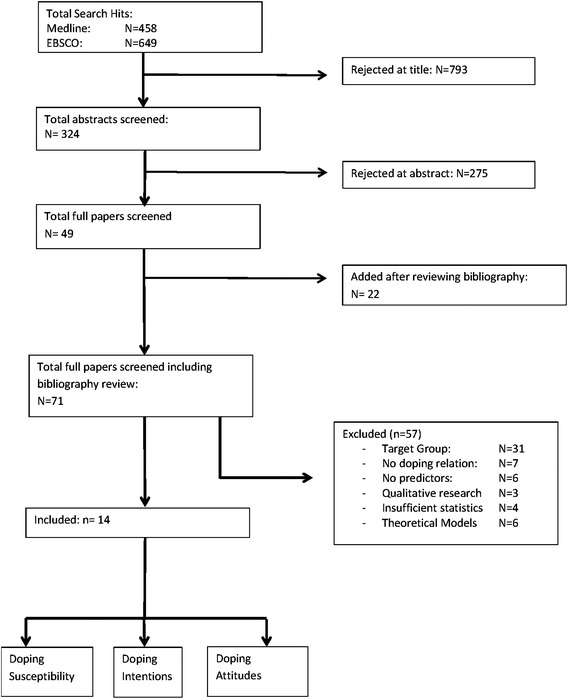
Flow chart of search strategy

Due to the meta-analytic approach, the review was limited to studies that evaluated predicting factors with respect to doping intentions, susceptibility, and/or behaviour of elite athletes competing on national level and above. We excluded manuscripts in which the sample was described with words such as “non-competitive”, “amateur” and/or “competing at club level”. Studies with focus on no matter which age groups, type of sports, and country were included. Studies that were not aimed at evaluating predictors with respect to intentions, susceptibility, and/or behaviour but rather included findings of this kind as ancillary results were excluded. Studies that focused on body building, gym-users and high-school/college sports were excluded, especially given the evidence of different reasoning (i.e., body image) for taking prohibited substances within these sports (Laure et al. 2004; Leifman et al. 2011). Studies that focused on adolescents participating in amateur level (i.e. high-school sport) were excluded. Publications that reported results from a mix of elite and non-elite athletes as per definition of the authors were excluded if such results were undistinguishable. Studies not reporting Pearson’s correlation, odds ratio or the mean standardized difference were not included in the meta-analytical statistics but included in the study. Finally, reports that described solely theoretically developed models without empirical testing were excluded. Figure 1 summarizes the study search and inclusion process.

### Excluded articles

The study search yielded a total of 1107 results, and 793 of these studies were rejected based on the title. A total of 324 abstracts were screened, and the full text of 71 of these studies including the bibliography was analysed. In sum, 57 articles were rejected due to the target group (31), no doping relation (7), no predictors (6), qualitative research (3), no report on Pearson’s correlation, odds ratio or the mean standardized difference (4), or description of theoretical models (6).

### Included articles

In total, 14 studies were included in the analyses. Four studies focused on doping intentions (Barkoukis et al. 2013, 2015; Lazuras et al. 2010, 2015), four studies focused on doping susceptibility (Barkoukis et al. 2014; Gucciardi et al. 2011; Hodge et al. 2013; Whitaker et al. 2014), six studies focused on doping behaviour (Donahue et al. 2006; Dunn and Thomas 2012; Jalleh et al. 2014; Mazanov et al. 2008; Petroczi 2007; Uvacsek et al. 2011).

### Quality analyses

Based on the nature of research in doping prevention, most studies are either quantitative self-reporting or qualitative interviews. Until now, barely any randomized controlled trials and/or experiments have been performed to evaluate predictors of doping attitudes, intentions, susceptibility, and/or behaviour. Therefore, applying methods such as the scale developed by Jadad et al. (1996) for data quality analysis was not feasible; however, we evaluated data in terms of their quality based on sample size, response rate, reliability (Cronbach’s alpha), and comparability of questionnaires.

### Data synthesis

To structure the outcomes with respect to different psychological concepts, we aligned the organization to psychological constructs from the literature. Therefore, attitudes, subjective norms, perceived behavioural control (PBC), and intentions were subsumed under the concept of TPB (Ajzen 1991). (Non-) user favourability and (non-) user similarity were subsumed under the construct of prototype modelling used by Whitaker et al. (2014), and additionally, we added descriptive norms to this construct (operationalized in all of the included studies as the estimated prevalence of doping in others) (Barkoukis et al. 2013, 2014, 2015; Lazuras et al. 2010, 2015; Uvacsek et al. 2011; Whitaker et al. 2014). As suggested by the theoretical drugs in sports deterrence model (DSDM) (Strelan and Boeckmann 2003), which is based on the deterrence theory, we subsumed situational temptation (as mostly pressure from the outside), personal morality, affordability, availability, legitimacy, threat, and benefit appraisal under the construct of deterrence theory (DT). Variables describing autonomous/intrinsic, controlled/extrinsic, and amotivation as well as coach-controlled, controlling teammate, autonomy-supportive coach, and teammate climate were combined under the concept of self-determination theory (SDT) (Ryan and Deci 2000). Variables of sport motivation (win orientation, competitiveness, goal orientation, mastery avoidance/effort, performance avoidance/ability, and performance approach/external reasons) were aggregated under the construct of achievement goal orientation/sport orientation (AGO) (Gill and Deeter 1988). Sportspersonship was the term used for the sportspersonship orientation scale, which was developed by Vallerand et al. (1997) and includes items such as respect for rules, opponents, and officials. Any kind of moral operationalization was also subsumed under sportspersonship. Experience was the term used for the combination of knowledge, past and current behaviour.

As effect sizes, Pearson’s correlation coefficients were given for the examination of the relation of two continuous variables and odds ratios (OR) for the examination of the relation of two dichotomous variables, respectively. Whenever different operationalization methods were applied for the same concept in different studies, i.e. both, dichotomous and continuous variables were used for the same construct in different studies, the OR or the mean standardized difference was converted to Pearson’s correlation to allow comparison of the studies. This procedure was previously proposed by Borenstein et al. (2011). According to Schmidt and Hunter (2015), random-effect models in meta-analysis of correlations are superior to fixed-effect models in terms of the accuracy of the confidence intervals. Therefore, meta-analytic methods were applied according to the random-effect method of Hedges and Olkin (1985), described in detail by Field (2005).

The mean effect size for every predictor was calculated using weighted Fisher’s r-to-Z and Z-to-r transformation for Pearson’s correlations (Fisher 1921). Due to the small number of included studies, this method is less biased than other methods (e.g. Hunter & Schmidt method) (Field 2005). Additionally, 95 % confidence intervals were calculated for the mean effect sizes, when possible. Pearson’s correlations between 0 and 0.1 were considered as small, 0.1 and 0.3 as medium and 0.3 and 0.5 as large (Cohen 2013). The classification of OR was defined as small (1.68–3.47), medium (3.47–6.71) and large (>6.71) (Chen et al. 2010).

## Results

### Methodological quality of included studies

Available Cronbach’s alpha ranged between 0.51 and 0.98. After discussion among the authors, no study was excluded due to quality issues, even though Cronbachs-α was not indicated for all studies. Different operationalization methods of the included constructs are outlined in Table 1.

**Table 1:** Operationalization analyses

Construct	Questions	Cronbach’s α	Author
Achievement goal theory	Approach and Avoidance Achievement Goal Questionnaire (Conroy et al. 2003)^a^	0.73–0.80	Barkoukis et al. (2013)
	Approach and Avoidance Achievement Goal Questionnaire (Conroy et al. 2003)	0.73–0.80	Barkoukis et al. (2014)
	Approach and Avoidance Achievement Goal Questionnaire (Conroy et al. 2003)^a^	0.73–0.80	Lazuras et al. (2015)
Affordability anticipated regret	Single-item: semantic differential^a^: expensive-cheap	0.93	Jalleh et al. (2014)
	Four items^a^: “If I use PES to enhance my performance during this season, I will: regret it/be disappointed/feel bad/feel shame	–	Barkoukis et al. (2015)
Attitudes/outcome expectancies	(a) Indication of the importance of that sport having an effective testing program^a^ (b) belief a doping problem exists in their sport^a^		Mazanov et al. (2008)
	Five items^a^: help compensate time loss after injury, help athletes in sport situations, risk is exaggerated, unavoidable in competitive sport, no difference in doping and technical support (e.g., hypoxia)		Gucciardi et al. (2011)
	Four semantic differentials^a^: “The use of performance enhancing substances (PES) is…”: good/useful/beneficial/ethical	0.77	Lazuras et al. (2010)
	Four semantic differentials^a^: “The use of performance enhancing substances (PES) is…”: good/useful/beneficial/ethical	0.74	Lazuras et al. (2015)
	Four semantic differentials^a^: “The use of PES is…”: good/useful/beneficial/ethical	0.77	Barkoukis et al. (2013)
	Four semantic differentials^a^: “The use of PES is…”: good/useful/beneficial/ethical	–	Barkoukis et al. (2014)
	Four semantic differentials^a^: “The use of PES is…”: good/useful/right/beneficial	–	Barkoukis et al. (2015)
	PEAS (Petróczi and Aidman 2009)	0.83	Petroczi (2007)
	PEAS (Petróczi and Aidman 2009)		Uvacsek et al. (2011)
	PEAS (Petróczi and Aidman 2009)		Whitaker et al. (2014)
	PEAS (Petróczi and Aidman 2009)	–	Hodge et al. (2013)
	Two items^a^: Need to use PES to perform at high level, consideration of an offer of PES		Jalleh et al. (2014)
	14 items^a^: agreement to statements to potential negative and positive outcomes		Whitaker et al. (2014)
Availability	Single-item: semantic differential^a^: impossible-easy to buy	0.90	Jalleh et al. (2014)
Beliefs about causes of success in sport	BACCS-Questionnaire (Duda and Nicholls 1992)^a^ 4 factors: motivation-effort reflecting attribution of success during task involvement, normative attributing success to ability, deception: attributing success to illegitimate behaviour, external factors	–	Barkoukis (2013)
Benefit appraisal	Two items^a,b^: Impact of PES on performance, rewards for performing well	0.87	Jalleh et al. (2014)
	Single-item: How necessary is it to compete at best level		Gucciardi et al. (2011)
Descriptive norm	Projected use in others		Uvacsek et al. (2011)
	Single-item: perceived prevalence in others		Lazuras et al. (2010)
	Three distinct items^a^: perceived prevalence of (a) athletes competing at the same level, (b) in their sport, and (c) peer athletes		Lazuras et al. (2015)
	Single-open-ended question^a^: out of 100 %, how many elite athletes in Greece…	–	Barkoukis et al. (2013)
	Three open-ended questions about beliefs of other athletes’ doping: out of 100 %, how many elite athletes…	–	Barkoukis et al. (2014)
	Two open-ended questions: e.g. out of 100 %, how many athletes at the same to you level do you think engage in doping to enhance their performance?	–	Barkoukis et al. (2015)
	Two items^a^: Of the athletes you know, how many use PES…, the four of the athletes you know best, how many use PES…		Whitaker et al. (2014)
	Single-item: Expression of presumed opinion regarding doping use	0.94	Petroczi (2007)
Intentions	Two items^b^: Situation to have been tested in- and out of competition		Mazanov et al. (2008)
	Three items^a^: I intend to use PES to enhance my performance during this season	0.97	Barkoukis et al. (2013)
	Three items^a^: I intend to use PES to enhance my performance during this season		Barkoukis et al. (2015)
	Three items reflecting perceived likelihood to engage in PES use: Two items of self-predictions and one item of self-prediction of substances that are cheap, hard to detect and with great effects	0.81	Lazuras et al. (2015)
Knowledge	5 items^b^: substances and methods 5 items^b^: testing procedures 5 items^b^: rights and responsibilities 4 items^b^: sanctions		Mazanov et al. (2008)
Legitimacy	Five items^a^: Security of testing procedure, equal treatment; fair hearing on positive appeal; fair hearing before sanctions, fair hearing in CAS		Jalleh et al. (2014)
	Three items^a^: Security of sampling; seriousness of preventive approaches; effectiveness of NADA		Gucciardi et al. (2011)
	Short form of MDSS^a^ (Boardley and Kavussanu 2008)		Hodge et al. (2013)
	Three items: I would cheat if it helps me win; if others are cheating, I think I can too; I would cheat if I can get away with it		Gucciardi et al. (2011)
	Two items^a^: Moral judgment; moral emotions	0.88	Jalleh et al. (2014)
	Three items^a^: e.g. Doping use is against my moral principles		Barkoukis et al. (2015)
Motivational climate	2 constructs: (a) autonomy supportive coach/teammate: adapted from health care climate questionnaire (Williams et al. 1999) (b) controlling coach/teammate: Coach controlling behaviour scale (Bartholomew et al. 2010)		Hodge et al. (2013)
Past use/current use	7 items (never, briefly, moderately, still think about it, briefly used, occasionally used, regularly use)		Gucciardi et al. (2011)
	Do you use…		Mazanov et al. (2008)
	Singe-item^a^: Have you ever used PES…		Barkoukis et al. (2013)
	Single-item^a^: Have you ever used PES…		Lazuras et al. (2010)
	15 items (banned by IOC)^a^: Have you used…	0.92	Donahue et al. (2006)
	Two items^a^: I feel in complete control over whether I will use PES…	0.76	Lazuras et al. (2010)
	Single item^a^: how frequently do you use PES to improve your performance?	–	Lazuras et al. (2015)
	Two items^a^: I feel in complete control over whether I will use PES…(Self-esteem: PBC + Situational temptation)	0.76	Barkoukis et al. (2013)
	Two items^a^: I feel in complete control over whether I will use PES		Barkoukis et al. (2015)
Personality	Risk taking^a^	0.75	Jalleh et al. (2014)
Prototype perception	Four items: Favourability of perceived user/non-user, compliance perceived characteristics of users/non-user with own characteristics	–	Whitaker et al. (2014)
Self-Determination	Sport Motivation Scale (Pelletier et al. 1995)^a^: intrinsic, extrinsic, amotivation	0.67–0.87	Lazuras et al. (2015)
	Sport Motivation Scale (Pelletier et al. 1995)^a^: intrinsic, extrinsic, amotivation	0.67–0.87	Barkoukis et al. (2013)
	Two subscales from Sport Motivation Scale (Brière et al. 1995)^a^: extrinsic and intrinsic motivation	0.67–0.73	Donahue et al. (2006)
	Sport orientation questionnaire	0.96–0.98	Petroczi (2007)
Self-efficacy/PBC	Personal competencies^a^: three items (e.g. I feel in complete control over whether I will use PES to enhance my performance during this season)	0.72	Lazuras et al. (2015)
Self-esteem	Four items^a^: As worth as much as other people, ability as other people, positive attitude towards oneself, satisfied with oneself	–	Gucciardi et al. (2011)
Situational temptation/doping susceptibility	Four items^a^: How much would you be tempted to use PES if…: coach suggests, most colleagues of yours are using, prepare for important competition, told to enhance performance	–	Barkoukis et al. (2014)
	Four items^a^: How much would you be tempted if…: coach suggests, belief that colleagues use, told to enhance performance, prepare for competition	0.86	Barkoukis et al. (2013)
	Four items^a^: How much would be tempted if…: coach suggests, belief that colleagues use, told to enhance performance, prepare for competition	0.86	Lazuras et al. (2010)
	Five items^a^: How much would you be tempted if…: coach suggests, belief that colleagues use, told to enhance performance, prepare for competition, feeling disadvantaged	0.85	Lazuras et al. (2015)
Sportspersonship	Multidimensional Sportpersonship Orientation Scale (Vallerand et al. 1997)^a^	0.51–0.83	Barkoukis et al. (2013)
	Two subscales from the Multidimensional Sportpersonship Orientation Scale (Vallerand et al. 1997)^a^: “respect and concern for the rules and officials”, “social conventions”	0.83–0.86	Donahue et al. (2006)
	Fours subscales from the Multidimensional Sportpersonship Orientation Scale (Vallerand et al. 1997)^a^:	0.71–0.90	
Subjective norms	Four items^a^: coach/doctor/fellow/family would approve of using PES	–	Whitaker et al. (2014)
	Single-item (reference group)^a^: How much would it be stopping you from using PES if you think about what other think of you using PES	–	Gucciardi et al. (2011)
	Single-item^a^: Reference group (moral judgment of reference group)	0.74	Jalleh et al. (2014)
	Three items^a^: Most people who are important to me would want me to use PES to enhance my performance	0.84	Barkoukis et al. (2013)
	Three items^a^: Most people who are important to me would want me to use PES to enhance my performance		Barkoukis et al. (2015)
	Three items^a^: People would want me to…	0.84	Lazuras et al. (2010)
	Three items^a^: People would want me to…	0.81	Lazuras et al. (2015)
	Three items^a^: Most people important to me would want me to use PES…	–	Barkoukis et al. (2013)
	How much consideration would you give the offer if: under medical supervision, no/low financial cost, significant difference in performance, currently not detectable	–	Hodge et al. (2013)
Susceptibility	Four items^a^: threat to health (once vs. regular use), deterrence in competition (detection), deterrence out of competition	0.87–0.91	Jalleh et al. (2014)
Threat appraisal	Three items^a^: How likely to get away with it (in and out of competition), how likely is a successful appeal?	–	Gucciardi et al. (2011)
Willingness to dope	10 scenarios (e.g., you suffer a dip in performance…) how willing would you be to use PES^a^	–	Whitaker et al. (2014)

*PEAS* Performance Enhancement Attitude Scale, *N/A* not available, *PBC* perceived behavioural control

^a^Likert scale

^b^Nominal scale

### Study participants

Basic characteristics of the included studies are displayed in Table 2. Three studies focused on adolescent athletes (Barkoukis et al. 2014; Donahue et al. 2006; Lazuras et al. 2015). Sample sizes ranged from 60 (Barkoukis et al. 2015) to 1684 (Dunn and Thomas 2012). Performance enhancing substance use prevalence in the included studies ranged from 4 % (Mazanov et al. 2008) to 14.6 % (Uvacsek et al. 2011).

**Table 2:** Basic characteristics of included studies

Authors	Year	Country	Target population		Outcome variable	Statistical analysis	R^2^*	Psychological concepts	SD
			N	Age ± SD					
Barkoukis et al.	2013^a^	Greece	673 (750)	25 ± 5.89	(I) Intentions Lifetime never dopers	Hierarchical Regression	41.2 %	SDT, AGT, sportspersonship, TPB, PM (part)	No
			74 (750)		(II) Intentions Lifetime ever dopers	Hierarchical Regression	78.2 %		
Barkoukis et al.	2014	Greece	309	16.64 ± 1.15	Susceptibility	Hierarchical Linear Regression	42.2 %	AGT, TPB (part), DT	No
Barkoukis et al.	2015	Greece	60	–	Intentions	Hierarchical Regression	67 %	TPB	
Donahue et al.	2006	Canada	1.201	16.34 ± 2.43	Behaviour	SEM	–	SDT, sportspersonship, use	Yes
Dunn and Thomas	2012	Australia	1.684	22 ± 4.0	Behaviour	Binary Logistic Regression Model	30 %^b^	DT, PM (part)	No
Gucciardi et al.	2011	Australia	670	23.75 ± 8.49	Susceptibility	SEM	11 %	TPB (part), DT, morality, personality	No
Hodge et al.	2013	New Zealand	224	20.3 ± 3.1	Susceptibility	SEM	22 %	SDT^c^, MDE, TPB (parts)	No
Jalleh et al.	2014	Australia	1237	23 ± 7.8	Behaviour	SEM	13 %	TPB (part), DT, morality, personality	No
Lazuras et al.	2010	Greece	1075	25 ± 5.89	Intentions	Hierarchical Regression analysis	69.2 %	TPB, PM (part), DT	Yes
Lazuras et al.	2015	Greece	816	16.08 ± 1.50	Intentions	Hierarchical Regression analysis	57.2 %	TBP, SDT, AGT, sportperonship, norms, self-efficacy (PBC + situational temptation)	Yes
Mazanov et al.	2008	UK	757	18–23	Behaviour	Logistic regression	–	TPB (part), experience	No
Petroczi	2007	USA	199	20.2 ± 2.15	Behaviour	SEM	–	SOT (GOT), beliefs, TPB (part), experience	Yes
Uvasczek et al.	2011	Hungary	82	21.43 ± 2.82	Behaviour	Multivariate regression model	–	Experience, PM (part), TPB (part)	No
Whitaker et al.	2014	UK	729	28.8 ± 10.1	Susceptibility	Multivariate regression model	54.4 %	PM, TPB (part), experience, expectancies	Yes

Sample with mixed target population → only results of elite athletes entered this table

*AGT* achievement goal theory, *CSA* covariance structure analysis, *DN* descriptive norm, *DT* deterrence theory, *GOT* goal orientation theory, *MDE* moral disengagement, *N/A* not applicable, *PBC* perceived behavioural control, *PM* prototype modelling, *SD* socially desirable behaviour, *SDT* self-determination theory, *SEM* structural equation model, *SO* sport orientation, *TBP* theory of planned behaviour

* *p* < 0.05

^a^Study population was combined again for meta-analytic calculations

^b^Nagelkerkes R^2^

^c^SDT extended by environment

### Outcome measures

All studies included only one outcome variable of interest: (a) intentions (Barkoukis et al. 2013, 2015; Lazuras et al. 2010, 2015), (b) susceptibility (Barkoukis et al. 2014; Gucciardi et al. 2011; Hodge et al. 2013; Whitaker et al. 2014) and (c) behaviour (Donahue et al. 2006; Dunn and Thomas 2012; Jalleh et al. 2014; Mazanov et al. 2008; Petroczi 2007; Uvacsek et al. 2011). Total variances explained are displayed in Table 2. Four studies did not indicate explained variance values (Donahue et al. 2006; Mazanov et al. 2008; Petroczi 2007; Uvacsek et al. 2011). Social desirable behaviour was controlled for in five studies (Barkoukis et al. 2014; Lazuras et al. 2010, 2015; Petroczi 2007; Whitaker et al. 2014). Significant predictors for doping intentions, susceptibility, and behaviour including the respective effect sizes are displayed in Table 3. None of the studies directly addressed contextual or sporting culture-related predictors. Yet, based on the operationalization (refer to Table 1) predictors subsumed under situational temptation, motivational climate, subjective norms, and the self-determination theory might represent facets of sporting culture (Stewart and Smith 2008).

**Table 3:** Results of meta-analysis predicting doping intentions, susceptibility and behaviour

Outcome	Predictor	k	n	Effect size	95 % CI lb	95 % CI ub
Doping intentions	Situational temptations^c^	4	2535	0.72^a^	0.69	0.75
	Attitudes	4	2535	0.60^a^	0.56	0.63
	Subjective norms^c^	4	2535	0.50^a^	0.45	0.55
	Descriptive norms	1	650	0.17^a^	0.09	0.24
	PBC	3	1885	0.27^a^	0.20	0.33
	Past behaviour	1	1075	0.29^a^	0.23	0.34
	Mastery avoidance	1	750	0.09^a^	0.02	0.16
	Anticipated regret	1	650	−0.48^a^	−0.54	−0.42
Doping susceptibility	Attitudes	4	1905	0.47^a^	0.32	0.59
	Subjective norms^c^	2	1038	0.48^a^	0.39	0.57
	Current behaviour	1	729	0.54^a^	0.48	0.58
	User favourability	1	729	0.50^a^	0.44	0.55
	User similarity	1	729	0.49^a^	0.43	0.54
	Competition level	1	729	−0.15^a^	−0.22	−0.08
	Mastery approach/deception	1	309	0.29^a^	0.18	0.39
	Descriptive norms	1	309	0.18^a^	0.07	0.29
	MDE	1	224	0.25^a^	0.12	0.37
	Controlling coach climate^c^	1	224	0.20^a^	0.07	0.32
Doping behaviour	Attitudes	3	2,076	0.30^a^	0.16	0.42
	Sportspersonship	1	1201	−0.23^a^	−0.28	−0.18
	SDT (only extrinsic)^c^	1	1201	0.11^a^	0.05	0.17
	Situational temptation (availability)^c^	1	974	17.1^b^	8.4	34.9
	Descriptive norms	1	974	3.0^b^	2.0	4.7
	Age (≤18 years)	1	757	4.27^b^	n.a.	n.a.
	Gender (male)	1	757	1.65^b^	n.a.	n.a.
	Knowledge (better)	1	757	1.19^b^	n.a.	n.a.
	Doping beliefs	1	199	0.40^a^	0.28	0.51
	Current behaviour	1	72	0.26^a^	0.03	0.47

k: number of studies, n: total sample, 95 % CI lb: 95 % confidence interval lower bound, 95 % CI ub: 95 % confidence interval upper bound

*PBC* perceived behavioural control, *MDE* moral disengagement, *SDT* self-determination theory, *n.a.* not applicable

^a^Pearson’s correlation r

^b^Odds ratio

^c^Represents facets of sporting culture based on its operationalization

## Discussion

The major aims of this study were first to critically review published findings regarding predictors of doping intentions, susceptibility, and behaviour and second to analyse and discuss to what extent these results also include factors from beyond the athlete-centred, psychological perspective. Findings outlined that barely any quantitative research included variables beyond an athlete-centred focus and most of the included studies stem from socio-psychological approaches. In regard to the investigated variables, situational temptation and attitudes were the strongest positive predictors for doping intentions, whereas current behaviour and subjective norms were the strongest predictors for doping susceptibility. Furthermore, doping behaviour was best predicted by situational temptation followed by attitudes and doping beliefs.

For all three outcome variables, our study confirms most of the results found by Ntoumanis et al. (2014). However, in contrast to the Ntoumanis et al. (2014) study, we found that in elite athletes competing in national international competitions and adherent to the WADA code, attitudes, but not intentions, were a predictive factor for doping behaviour. In the literature, attitudes were the most proximal antecedent to doping behaviour among studies that excluded intentions (Jalleh et al. 2014). In line, our meta-analysis also outlined a positive predictive value of attitudes on behaviour. Summarizing the results from previous anti-doping research, it appears that in the framework of doping, attitudes do have a positive predicting value even though this association is controversially discussed in literature. Ajzen and Fishbein (1977), the founder of the theory of planned behaviour concluded that the attitude-behaviour relationship may be weak and inconsistent. Also in the doping prevention literature findings of the individual studies are inconsistent, which might be explained by methodological heterogeneity in the operationalization of attitudes.

In contrast to the study of Ntoumanis et al. (2014) and to previous research from the theory of planned behaviour (Armitage and Conner 2001; Bilic 2005; Elliot et al. 2003; Godin and Kok 1996), intentions were no predictor for doping behaviour. An explanation might be found in the ongoing discussion about whether doping behaviour is considered to be rational and volitional decision making. For example, Petroczi and Aidman (2008) highlighted the fact that doping occurs in a life-cycle model in which individual differences, as well as systemic factors (e.g., motivational climate), play vital roles in self-belief formation (i.e., attitudes and intentions) and interact with situational and environmental factors (e.g., experience, perceived behavioural control, availability) to influence doping behaviour. Consistently with this hypothesis, the sporting culture, which is said to shape attitudes, beliefs and intentions (Smith et al. 2010), is considered to be another important factor in the decision to dope (Copeland and Potwarka 2016; Engelberg et al. 2015).

This argument leads to the second major aim of this meta-analytic review to analyse aspects beyond the athlete-centred approaches. None of the reviewed studies neither explicitly included macro-level factors such as sporting culture and context nor have any of them based their rationale on sociological theories. This finding is interesting as qualitative research suggests the usefulness of macro-level factors in explaining doping behaviour. Some facets of the included variables are part of this macro level and might help to understand why athletes dope and other do not, even though they might display similar personality traits and characteristics. In detail, we found self-determination theory, descriptive norms, moral and situational temptation to be significant predictors for doping behavior and controlled coach climate and subjective norms were predictors for doping susceptibility.

Given the findings from qualitative approaches, especially the sporting culture seems to be a very powerful link in the process of the decision to dope (Copeland and Potwarka 2016; Engelberg et al. 2015; Smith et al. 2010). This is explained based on the social ecological theory that considers attitudes and behaviours to be not only driven by personal intrinsic factors but also by environmental influences. These also include the proximal athletes’ network and the significant role of coaches, team-mates and also peers that has been previously reported elsewhere (Madigan et al. 2016; Martin et al. 2014; Ommundsen et al. 2006). Being part of a certain sporting culture might also mean adopting certain norms and beliefs the individual might not have had prior to entering this specific culture. Especially in cycling, the majority reports a culture that represents a “normalization of doping” (Pappa and Kennedy 2012). Not living up to these standards might threaten the social identity of the athletes, which is why these athletes might decide to dope. Apparently, it is not enough to understand that norms impact on doping behaviour but it is important to also understand how these norms are constructed (Ohl et al. 2015). Sporting culture does not only shape norms but also moral understanding on what is right or wrong. The sporting culture of elite sport also defines a certain competitive nature that is posed on the athletes (Stewart and Smith 2008). Stewart and Smith (2008) argue further that such a sporting culture might result in violating aspects of autonomy and self-determination, factors that we could also identify to be associated with doping behaviour.

Given the apparently great influence coaches seem to be able to execute on athletes (Huybers and Mazanov 2012) in combination with the predictive value of coach climate on doping susceptibility, we also support the call for broader value-based education that includes athletes’ support personnel (Momaya et al. 2015). However, coaches do not consider doping prevention as their task (Engelberg and Moston 2016). As indicated by Copeland and Potwarka (2016), these preventive approaches should ensure the improvement of ethical team culture by including leadership elements and informing athletes about the actual prevalence of doping, which should prevent a “normalization-of-doping” culture as proposed by Woolf et al. (2014).

Major concerns remain about the methodological approaches used in socio-psychological research studies on doping behaviour that have emerged over the past decade. Future studies should focus on using reliable and comparable instruments for data aggregation as suggested by Backhouse et al. (2007). Future research might also want to use instruments that are designed to enable the production of information on causality, such as experimental or longitudinal studies with at least two time points. Before investing financial resources in prevention measures, we should try to identify intervention points that have proven to be causal for doping behaviour throughout various studies, and also develop and evaluate new intervention measures. Last but not least, missing links between intentions and behaviour might be explained with (a) the difficulty to reliably assess doping behaviour using questionnaires and (b) because perspectives beyond the athlete-centred psychological one should be integrated. Other approaches, as for example shown in the (sports) economics literature based on the game theory (Buechel et al. 2014; Haugen 2004) analyse the doping phenomenon in sports from a socio-economic perspective. The integration of these economic theories, such as Nash equilibrium, Pareto efficiency and the prisoner’s dilemma would be also interesting for this study. Nevertheless, including this perspective would be beyond the scope of this meta-analysis, but should be included inpotential further research.

Some limitations of the present review need to be addressed as well. Socially desirable behaviour was controlled in only five studies (Barkoukis et al. 2014; Lazuras et al. 2010, 2015; Petroczi 2007; Whitaker et al. 2014), and the overall impact of socially desirable behaviour was small. Nevertheless, we cannot exclude potential biased results in studies that (a) did not control for socially desirable behaviour and (b) due to the fact that its effect on model level might be more significant (Petroczi and Nepusz 2011). All included studies that reported on doping behaviour measured behaviour via self-report rather than objective measures (e.g., hair analyses, etc.). Therefore, the potential for inaccurate self-reports may lead to biased results. A possible solution to this problem is offered by using either experimental designs or randomized response techniques as used in previous research (de Hon et al. 2015; Pitsch and Emrich 2011). Future research should assess predictors of doping behaviour by assessing the dependent variable using such techniques. Another limitation might be the diverse definitions of “performance enhancing substances (PES)”. Not all of the included studies presented a PES definition to their respondents. This might have led to under- and overestimation of the figures in the original studies, as not all respondents might be correctly informed about whether or not substances they consume are PES as defined by the WADC. Finally, ecological validity may be an additional limitation of the present review. Included studies were restricted to a narrow target population of elite athletes, and further research is needed to validate these results in additional target samples. Furthermore, it would be interesting to compare results between different definitions of “elite” as proposed by Swann et al. (2015).

## Conclusion

In summary, aggregating previous research resulted in some generalizable predictors of doping intention, susceptibility and behaviour emerged from this review. However, most of these predictors are athlete-centred and ignore macro-level factors that might help to explain how certain individual traits are developed and why some theoretical models from health-psychology do not seem to be easily transferred to doping behaviour. This finding is astonishing, as results from qualitative approaches suggest the usefulness of macro-level factors in explaining doping behaviour. These findings must be acknowledged as a lot of the qualitative approaches included and questioned athletes who have committed to doping and therewith overcome the biggest limitation of the quantitative studies, namely reliably assessing doping behaviour. An additional striking factor is the limited data availability from only six Western countries. Given this limitation, no conclusion can be drawn about potential cultural factors that impact on the decision to dope.

The findings from this review call for a critical discussion of whether current doping-prevention research needs to take new directions. These strategies are often aimed at changing attitudes and intentions because health-related behavioural theories suggest its effectiveness. However, findings of the current study indicate an unclear picture about the predicting value of intentions on doping behaviour. This might be due to methodological issues or the fact that doping behaviour is too complex and macro level factors must not be ignored. Obviously, psychologists and sociologists both do a very important job in explaining doping behaviour. Yet their findings need to be integrated, as it appears that doping behaviour cannot be explained by ignoring the one or the other. Literature that directly addresses the macro level is mainly narrative and based on qualitative research, which is a useful tool to receive in depth-information on an unknown field but possibly not suitable to draw general conclusions that are sound enough to base prevention strategies on. Thus, it allows generating hypotheses about why athletes dope that need to be integrated in future quantitative approaches to test for its external validity. Finally, there is the urgent need for sound and qualitatively high studies about the mechanisms behind the decision to dope also from Eastern countries to assess possible specific cultural characteristics.
